# Clinical validation of the Unesp-Botucatu acute pain scale in sheep undergoing orthopedic surgery

**DOI:** 10.1371/journal.pone.0323132

**Published:** 2025-05-12

**Authors:** Nuno Emanuel de Oliveira Figueiredo da Silva, Pedro Henrique Esteves Trindade, Gustavo Venâncio da Silva, Flávia Augusta de Oliveira, Marilda Onghero Taffarel, Mayara Travalini de Lima, Rubia Mitalli Tomacheuski, Gustavo dos Santos Rosa, Ana Liz Garcia Alves, Stelio Pacca Loureiro Luna

**Affiliations:** 1 Department of Veterinary Surgery and Animal Reproduction, School of Veterinary Medicine and Animal Science, São Paulo State University (Unesp), Botucatu, Brazil; 2 Department of Large Animal Clinical Sciences, College of Veterinary Medicine, Michigan State University, East Lansing, Michigan, United States of America; 3 Department of Anesthesiology, Botucatu Medical School, São Paulo State University (Unesp), Botucatu, São Paulo, Brazil; 4 University Veterinary Clinic, School of Veterinary Medicine and Animal Science, Federal University of Northern Tocantins, Araguaína, Brazil; 5 Department of Veterinary Medicine, State University of Maringá, Umuarama, Paraná, Brazil; 6 Department of Population Health and Pathobiology, College of Veterinary Medicine, North Carolina State University (NCSU), Raleigh, North Carolina, United States of America; Research Institute for Brain and Blood Vessels, Akita Cerebrospinal and Cardiovascular Center, JAPAN

## Abstract

Unesp-Botucatu sheep acute pain scale (USAPS) was validated for assessing postoperative abdominal pain. We aimed to investigate the clinical applicability and test the psychometric properties of USAPS to assess postoperative pain in sheep submitted to orthopedic surgery. Twenty-three healthy sheep undergoing patellofemoral joint arthrotomy were video-recorded for three minutes before and after surgery, after postoperative analgesic rescue, and 24 hours post-surgery. Four evaluators, unaware of the recording time points, randomly assessed all videos twice at one-month intervals. Intra-observer reliability based on the Intraclass Correlation Coefficient (ICC) was very good for all evaluators (ICC: 0.82–0.93). Inter-observer reliability was very good for four of six pairs of evaluators (ICC: 0.84–0.9) and good for two (ICC: 0.77 and 0.80). Principal component analyses and confirmatory factor analysis confirmed the USAPS´s unidimensional structure. The concurrent criterion validity had a strong Spearman correlation (rho: 0.80) between the USAPS and the Visual Analogue Scale. Responsiveness was evidenced by the highest USAPS total score 2 and 24 hours after surgery, and intermediate scores after analgesic rescue. USAPS items had an acceptable Spearman item-total correlation (rho: 0.38–0.64), except appetite (rho: 0.25). Internal consistency was excellent according to Cronbach’s alpha (α: 0.84) and acceptable according to McDonald’s omega coefficients (ω: 0.75). Specificity was 100% and sensitivity was 71%. USAPS cut-off point was ≥ 4 of 10, the same applied for soft tissue surgery. The area under the curve of 0.91 demonstrates the high discriminatory capacity of the scale. The item appetite can be excluded without affecting the USAPS cut-off point. We concluded that USAPS had satisfactory psychometric properties and, is a valid and reliable clinical tool for assessing pain in sheep undergoing orthopedic surgery.

## Introduction

Recognizing and quantifying acute pain in sheep is challenging for veterinarians, farm workers, and researchers. Chilean sheep farmers recognize that castration and tail docking cause intense pain, however, 86–93% of them still do not use analgesics [1]. In Northern Ireland, 27% of sheep farmers did not use NSAIDs for pain relief [2].

Beyond use for food production, sheep are a relevant experimental model to investigate and treat human diseases [3]. In 2022, 15,909 sheep were used in European biomedical research [4] and their use has increased in Australia, New Zealand and the United States [5]. From 2014 to 2020 in Great Britain, sheep were used more frequently (1.8% of the total species) than pigs (0.4%) and cattle (0.2%) for research [6]. Mice, fish, rats and birds account for 92% of animals used in the European Union [4]. Sheep is a biomedical model for cardiovascular, orthopedic, reproduction, gene therapy, and neurodegenerative research to test new pharmacological agents and medical devices for clinical applications [5].

Sheep are a good model for translational orthopedic in vivo research in biomechanical (biomaterials and orthopedic implants), biochemical and histological processes of bone physiology because their weight, size, bone, joint structures and healing are similar to humans [7]. These analogies enable us to study numerous musculoskeletal pathologic conditions, such as the repair of fractures (23% of the studies were in sheep) and articular ligaments (19%), limb bone lengthening (9%), treatment of osteoarthrosis (8%) and osteoporosis (2%) and other injuries (39%) [8].

Creating valid species-specific scales to assess acute pain is essential for recognizing pain and determining the need for and effectiveness of analgesic treatment. Before the validation of the Unesp-Botucatu sheep acute pain scale (USAPS) [9] there were three main instruments available to assess acute pain in sheep. They were developed for 1) lambs submitted to orchiectomy and tail docking [10], 2) sheep implanted with ventricular assist devices [11] and 3) behavior evaluation protocol [12]. Recently, a systematic review [13], reported that only the USAPS [9] scored high for the strength of evidence of psychometric validation [13], according to the Consensus-based Standards for the Selection of Health Measurement Instruments (COSMIN) [14] in farm animals.

There are three scales to assess pain by using facial expression: 1) sheep with pododermatitis and mastitis (SPFES) [15], 2) Grimace scale in sheep after osteotomy [16] and the 3) Grimace scale in lambs after tail docking [17]. None of them met the COSMIN criteria to be classified as a high level of evidence and only SPFES showed a moderate level of evidence [18].

Although the psychometric properties of USAPS have been robustly validated to assess postoperative pain in sheep undergoing soft tissue surgery [9], USAPS still needs to be validated in other circumstances. Therefore, based on the use of sheep as a translational model, validation of USAPS for orthopedic surgery is demanded to ensure its versatility. In general, pre-existing tissue injuries leading to pain and greater surgical invasiveness degree are more frequent in animals submitted to orthopedic procedures compared to soft tissue surgery (e.g., laparotomy), requiring a more comprehensive analgesic strategy [19]. These differences may modify the expression of pain behaviors and the cut-off point.

This study aimed to investigate the clinical applicability and test the psychometric properties of USAPS to assess postoperative pain in sheep submitted to orthopedic surgery in addition to soft tissue (abdominal) pain. We hypothesized that USAPS shows a high strength of evidence of validation to assess acute pain after arthrotomy as reported for laparoscopy in sheep.

## Materials and methods

This opportunistic study was incorporated into the main project, “Use of heterologous fibrin sealant and mesenchymal stem cells in knee disorders”, approved by the Ethical Committee for the Use of Animals in Research of the School of Veterinary Medicine and Animal Science - São Paulo State University, Botucatu, São Paulo, Brazil - under the protocol 0111/2019. This blinded, randomized, prospective, and horizontal study followed the COSMIN guidelines [14], the Grading of Recommendations, Assessment, Development, and Evaluations (GRADE) [20], that grades the quality of evidence and the strength of its recommendations, checklist and terminology guidelines (S1 Table) to ensure methodological quality, and the Animal Research Reporting In Vivo Experiments (ARRIVE 2.0) [21] (S2 Table), adapted to the experimental design.

### Animals

We used 23 female Santa Ines sheep (*Ovis aries*) aged between 6 and 12 months (7.6 ± 2.2) and weighing between 29 and 37 kg (31.1 ± 6.90 kg). After deworming and vaccination on the University farm, the animals were allowed to adapt to the experimental environment for 14 days, where they were allocated in stalls (3.00 x 4.00 x 1.50 m - length x width x height) and kept in groups of four until the end of the experiment. The sheep had no previous knee injuries and underwent clinical examinations (mucosal colour, lymph nodes palpation, rectal temperature, rumen movements, heart, and respiratory rate), laboratory tests (complete blood count, biochemical and copro-parasitological exams), imaging tests (x-ray and ultrasound) and muscle biopsies to confirm their health. They were fasted for 24 hours on food and 12 hours on water.

### Anesthetic and analgesic protocol

One hour before surgery, one of the jugular veins was catheterized, and the sheep received 2.2 mg/kg of ceftiofur sodium (CEF 50^®^, Agener, São Paulo, Brazil) intramuscularly (IM) maintained daily for 10 days. Preanesthetic medication was carried out with 0.3 mg/kg of morphine (Dimorf^®^ 1%, Cristália, Itapira, SP, Brazil) and 0.2 mg/kg of midazolam (Dormonid^®^, Farmoquímica, Rio de Janeiro, RJ, Brazil) IM. Induction of anesthesia was performed with 3–5 mg/kg of propofol (Diprivan^®^, Aspen Pharma, Serra, ES, Brazil) until endotracheal intubation was possible. Inhalation anesthesia was delivered with isoflurane (Isoforine^®^, Cristália). Volume-controlled ventilation with a tidal volume of 10 ml/kg, inspired oxygen fraction of 40%, and inspiration/expiration ratio of 1:2 was used (Model 2800C Large Animal Anesthesia Ventilation System®). Peripheral hemoglobin oxygen saturation was monitored using a sensor positioned on the tongue, and 5 ml/kg/hour of lactate Ringer (RL physiological solution ^®^, Cristália) was infused intravenous (IV).

Sheep were subdivided into three echo-guided locoregional anesthesia techniques performed by the same anesthetist: 1 - lumbosacral epidural administration of 0.1mL/kg of bupivacaine (Neocaína^®^ 0.5%, Cristália); 2 – Infiltration between the popliteal artery and capsule of the knee (IPACK) combined with the adductor channel block of the saphenous nerve branch (0.3mL/kg of 0.5% bupivacaine) or 3 – adductor channel block of the sciatic and saphenous nerve branch (0.3mL/kg of 0.5% bupivacaine). Heart rate and rhythm were assessed by electrocardiography, invasive blood pressure was measured into the femoral artery, and expired end-tidal isoflurane concentration was adjusted to maintain mean arterial blood pressure between 60–100 mmHg (Datex Ohmeda Cardiopac 5®). Depth of anesthesia was assured by immobility, absence of palpebral reflexes, mandibular tone, and spontaneous respiratory efforts during mechanical ventilation. When the heart rate or mean arterial pressure rose above 20% of the values registered before surgery, transoperative analgesia was performed with 2.5 µg/kg of fentanyl (Fentanest^®^, Cristália) IV.

Knee arthrotomy with meniscus injury lasted around 1.5–2 hours. An arthrotomy of the left patellofemoral joint was performed. The patella was removed laterally, exposing the chondral surface of the medial femoral condyle, the trochlea, and the medial meniscus. Two lesions were performed: one in the medial meniscus and another at the distal end of the quadriceps muscle. After surgery, no cast was used, and sheep movement was not restricted.

### Data collection

**Fig 1:**
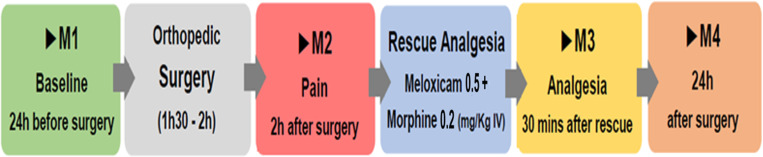
Timeline flowchart used for USAPS in the perioperative period of orthopedic surgery. USAPS: Unesp-Botucatu sheep acute pain scale.

Four animals were operated on each day. The procedures began at 09:00 am, and the last evaluation finished by 06:00 pm. The study was conducted from June to December 2019, and the average temperature and humidity of the environment varied between 12–28 ºC and 1–54%, respectively (https://pt.weatherspark.com). Geographical coordinates were: latitude 22º 54’ South; longitude 48º 29’ West and altitude 866 m (https://earth.google.com). An in-person researcher (NEOFS) filmed the animals for 3 minutes at each time point, with a digital camera (Gopro Hero5 Black®) positioned on a tripod. During this time, he scored USAPS. Recording time points were 24h before surgery without fasting (M1 – no pain), 2 h after surgery (M2 - pain), 30 minutes after analgesia (M3 - rescue), and 24h after surgery (M4 – 24h) (Fig 1). The postoperative analgesic intervention was conducted by the on-site evaluator, with 0.5 mg/kg of meloxicam (Maxicam^®^ 2%, Ourofino, Cravinhos, SP, Brazil) and 0.2 mg/kg morphine IV (Dimorf® 1%, Cristália), in sheep scoring ≥ 3 of 10 in USAPS [9]) 2h after surgery, immediately after recording M2 time point in the pen [this cut-off point was considered instead of ≥ 4 calculated for the soft tissue surgery validation study [9], because locomotion was not assessed]. The pain was reassessed after 30 minutes, and sheep that scored ≥ 3 of 10 in USAPS were treated with 25 mg/kg of metamizole (D500 Dipirona®, Zoetis, São Paulo, Brazil) IV. Then, after 30 minutes, if the pain score was still ≥ 3, sheep received a third analgesic rescue with 0.1 mg/kg of morphine IV and 1 mg/kg of ketamine (Cetamin®, Syntec; Santana de Parnaíba, SP, Brazil) IV and the pain was reassessed 15 minutes later. After the end of the experimental period, sheep were treated with meloxicam (0.5mg/kg) and metamizole (25 mg/kg) both IV for five to seven days. In case the USAPS pain score was ≥ 3, 0.1 mg/kg of morphine IV and 20 mg/kg of oral gabapentin were administered and repeated whenever necessary every 8 hours.

### Selection and training of evaluators

All evaluators were veterinary anesthesiologists with at least five years of experience evaluating pain scales in animals (FAO, MOF, MTL, and RMT). They were trained with videos demonstrating each behavior (www.animalpain.org) and were instructed on how to fill in the scale (Table 1). Next, they evaluated 10 videos of different pain intensities and after a week, the observers scored the same randomized videos. Because the intra-observer reliability was ≥ 80%, the evaluators were enrolled in the main study evaluation.

**Table 1:** Unesp-Botucatu sheep acute pain scale (USAPS) updated for orthopedic surgery excluding the locomotion item.

Item	Subitem (descriptors)	Score
**Interaction**	Active, attentive to the environment, interacts and/or follows other animals or sleeps.	0
	Apathetic: may remain close to other animals but interacts little.	1
	Very apathetic: isolated or not interacting with other animals, not interested in the environment.	2
**Locomotion**	Moves about freely, without altered locomotion; when stopped, the pelvic limbs are parallel to the thoracic limbs or sleeps.	0
	Moves about with restriction and/or short steps and/or pauses and/or lameness; when stopped, the thoracic or pelvic limbs may be more open and further back than normal.	1
	Difficulty and/or reluctant to get up and/or not moving and/or walking abnormally and/or limping; may lean against a surface.	2
**Head position** (consider the predominant time when the animal is not eating)	Head above the withers or eating or sleeping.	0
	Head at the height of the withers.	1
	Head below the withers.	2
**Posture** (occurrence of at least one episode of each behavior)	**A**. Arched back.	
	**B**. Extends the head and neck.	
	**C**. Lying down with head resting on the ground or close to the ground.	
	**D.** Moves the tail quickly and repeatedly (except when breastfeeding) and/or keeps the tail straight (except to defecate/urinate).	
	Absence of these behaviors.	0
	Presence of one of the related behaviors.	1
	Presence of two or more of the related behaviors.	2
**Activity**	Moves normally or sleeps.	0
	Restless, moves more than normal or lies down and gets up frequently.	1
	Moves less frequently or only when stimulated using a stick or does not move.	2
**Appetite**	Normorexia and/or rumination present.	0
	Hyporexia.	1
	Anorexia.	2

### Pain assessment and analgesic rescue

Four evaluators, unaware of the recording time points, watched and scored 86 [23 sheep in four (or 3) time points] randomized (for time points and animals) unedited videos of around three minutes. The videos’ order was randomized using the Microsoft Office Excel function = RANDOMBETWEEN (1;208). The evaluators scored about 21–22 videos weekly for one month (1^st^ phase). After a one-month interval, the evaluators rewatched the same videos in a new random order (2^nd^ phase). After observing each video, they answered if they would administer rescue analgesia (0 = no and 1 = yes), based on their clinical experience. Then, they assessed in half of the videos first the USAPS followed by the Visual Analogue Scale (VAS) and in the other half first VAS and then USAPS in a randomized order (VAS; a straight line 100 mm long, where “0” represents the animal without pain and “100” is the worst possible pain) [22].

### Statistical analysis

To estimate sampling size with the power analyses, it was considered that 80% of the animals undergoing surgery would present a score ≥ 4 after surgery (at M2) based on the USAPS cut-off point and none of them would present pain scores ≥ 4 before surgery (at M1). According to the chi-square test, 11 animals were needed for an alpha of 0.01 and a power of 90%.

Two data scientists (PHET e GVS) coordinated the statistical analysis using R software in the RStudio integrated development environment (version 4.1.0; 29 June 2021) [23].

Table 2 contains a detailed statistical description of each statistical method used to test the psychometric property of the USAPS.

**Table 2:** Statistical methods used for psychometrical testing of Unesp-Botucatu sheep acute pain scale (USAPS).

Type of analysis	Description	Statistical test
**Distribution** **of scores**	Frequency distribution of the presence of the scores ‘0’, ‘1’, and ‘2’ of each Unesp-Botucatu sheep acute pain scale (USAPS) item at each time point and in all time points grouped (MG).	Descriptive statistical analysis.
**Multiple association** **and dimensional structure**	Three steps defined the scale’s dimension structure: 1^st^ - Horn’s parallel analysis was used to decide the optimal number of principal components (dimensions) that should be retained; 2^nd^ - Principal components analysis (PCA) was conducted to analyze the multiple associations between the USAPS items in the main principal components indicated in the 1^st^ step and 3^rd^ - According to the confirmatory factor analysis parameters, comparative fit index (CFI), Tucker-Lewis index (TLI) and root mean square error of approximation (RMSEA) it was analyzed the mathematical dimensional structure of the scale.	An optimal number of principal components in the PCA (stats::princomp and factoextra::get_pca_var) was estimated based on Horn’s Parallel Analysis (psych::fa.parallel) [24]. Significant associations were considered when the loading value of each item was ≥ 0.50 or ≤ - 0.50. The confirmatory factor analysis parameters CFI, TLI (0–1, good > 0.90–0.94, excellent ≥ 0.95), and RMSEA (expecting values < 0.05) assessed the mathematical dimensional structure for the scale [25].
**Intra-observer reliability**	Repeatability - the level of agreement of each observer with themself was estimated by comparing the two phases of assessment, using the scores of each item and the total sum of the USAPS, visual analogue scale (VAS) and the indication for analgesic rescue. Analgesic rescue was indicated based on the evaluator’s experience answering the question before assessing the pain scales ‘Do you think it is necessary to provide analgesic rescue?’ yes (1) or no (0).	For the scores of each item of the USAPS and the indication for analgesic rescue, the weighted kappa coefficient (*k*_*w*_) was used; the disagreements were weighted according to their distance to the square of perfect agreement (biostatUZH::confIntKappa). The 95% confidence interval (CI) *k*_*w*_ based on 1001 replications by the bootstrap method was estimated (boot::boot.ci). For the sum of the USAPS, the intraclass correlation coefficient (ICC; irr::icc) was used, by applying the two-way mixed and alpha model, type consistency multiple. For VAS, the intraclass correlation coefficient (ICC; irr::icc) was used by applying the two-way mixed and alpha model, and type absolute agreement. Evaluators/measurements and their 95% CI based on 1001 replications, were calculated by the bootstrap method (boot::boot.ci). Interpretation of *k*_*w*_ and ICC: very good: 0.81–1.0; good: 0.61–0.80; moderate: 0.41–0.60; reasonable: 0.21–0.4; poor: < 0.2.[26,27]
**Inter-observer reliability**	Reproducibility – the level of agreement between the four evaluators using the scores for each item, the total sum of the USAPS, VAS, and the need for analgesic rescue.	
**Criterion** **validity**	1) Concurrent criterion validity (comparison with another instrument): the USAPS total score was correlated with the VAS	Spearman rank correlation coefficient (r_s_; Hmisc::rcorr) between the 2 scales. Interpretation of the degree of correlation rs (*p* < 0.05): < 0.20 very weak, 0.20–0.39 weak, 0.40–0.59 moderate, 0.60–0.79 strong and ≥ 0.80 very strong ([28,29])
	2) Concurrent criterion validity: the agreement between all evaluators (reproducibility).	See previous description for Inter-observer reliability: 1) Reproducibility.
	3) Predictive criterion validity was assessed by the number of sheep that should receive analgesic rescue according to the Youden Index (described below) in the time point of greatest pain (M2).	See sensitivity.
**Responsiveness**	The scores of each USAPS item and total score were compared over time, as well as the VAS score and indication of analgesic rescue (M1 vs. M2 vs. M3 vs. M4). Interpretation: differences in scores are expected to occur as follows: M2 ≥ M4 > M3 > M1	Responsiveness was conducted using a multilevel generalized linear model adjusted by negative binomial distribution (lme4::glmer.nb) applied to USAPS total score, a multilevel generalized linear model adjusted by Poisson distribution (lme4::glmer) applied to VAS score and to USAPS items and a multilevel binomial logistic regression (lme4::glmer) applied to sub-items of Posture item and to the indication of analgesic rescue. Time points, observers, and phases of assessment were included as explanatory variables, while individuals (sheep) were controlled as random variables in the model. Bonferroni test (lsmean::lsmeans and multcomp::cld) was used as a post-hoc test for multiple comparisons
**Construct validity** (determined by four methods)	1. Testing of three hypotheses: 1 - if the scale truly measures pain, the postoperative pain scores should be higher than the preoperative score (M2 > M1), 2 - the scores should decrease after analgesic rescue (M2 > M3) and 3 – the scores should increase again by 24h when the effect of analgesia should be fading	See Responsiveness
	2. Known-groups validity: pain-free animals (M1) should have lower pain scores than animals suffering pain (M2) and after analgesic rescue (M3).	See Responsiveness
	3. Internal relationships among items	See internal consistency, item-total correlation and PCA.
	4. Relationships with scores of other instruments.	See criterion validity.
**Item-total correlation**	The correlation of each item of the scale was estimated, and each item of USAPS was compared with its total score by excluding the evaluated item to analyze scale homogeneity, inflationary items, and relevance of each item on the scale.	Spearman rank correlation coefficient (r_s_; Hmisc::rcorr). Interpretation of correlation. *r*_*s:*_ Values between 0.3 and 0.7 were accepted [26].
**Internal consistency**	The interrelationship of the scores for each item on the scale was estimated. If each item was excluded and the internal consistency value decreased, it meant that items contributed to the total scale score.	Cronbach’s alpha coefficient (α; psy::cronbach) and McDonald’s omega coefficient (ω; psych::omega). Interpretation of α: 0.60–0.64 minimally acceptable, 0.65–0.69 acceptable, 0.70–0.74 good, 0.75–0.80 very good and > 0.80 excellent [30]. Interpretation of ω: 0.65–0.80 acceptable values and > 0.80 strong evidence [31]
**Specificity** **and** **Sensitivity**	Specificity: based on true negatives (TN) - animals without pain (score 0) at M1. Sensitivity: based on true positives (TP) - animals with pain (scores 1 or 2) at M2. For the total score of the scale, the percentage of animals that had scored below the cut-off point at M1 and equal or above the cut-off point at M2 was considered specificity and sensitivity, respectively.	$\begin{array}{c} SPM1=\frac{\text{TN}}{Total\;number},\;S\;M2=\frac{\text{TP}}{Total\;number}. \end{array}$ Specificity *(Sp)*, sensitivity *(S)* and its 95% CI were calculated according to the bootstrap method (epiR::epi.tests). Interpretation of specificity and sensitivity: excellent 95–100%, good 85–94.9%, moderate 70–84.9% and not specific or sensitive <70% [26].
**Optimal** **cut-off point** (determination of intervention score for analgesic rescue)	The time point (M1) of pain-free animals and M2 of the peak of pain were used to determine the optimal cut-off point. The indicative score for analgesic rescue was calculated by whether analgesia was indicated after the evaluators watched each video, according to their clinical experience (true values) and the total score of the scales as a predictive value to build a receiver operating characteristic (ROC) curve. The calculation of the area under the curve (AUC) indicates the discriminatory capacity (accuracy) of the test. The median of the score was used to estimate the optimal cut-off point and its confidence interval was used to estimate the diagnostic uncertainty zone. The cut-off point for analgesic rescue was determined based on the Youden index and its diagnostic uncertainty zone using all time points of pain assessment on the scales.	The ROC curve (pROC::roc and pROC::ci.sp’) and AUC graphically represent true positives (sensitivity) and false positives (1 -specificity). The Youden index (YI = [Sensitivity + Specificity] - 1), determined by the ROC curve consists of the simultaneous point of greatest sensitivity and specificity. The highest value of the Youden index represents the indication point for analgesic rescue: the optimal cut-off point. An AUC ≥ 0.90 indicates a high discriminatory capacity (accuracy) of the scale. The diagnostic uncertainty zone was determined by calculating the 95% confidence interval (CI) by replicating the original ROC curve 1001 times using the bootstrap method (pROC::ci.coords and pROC::ci.auc) [32,33].

USAPS - Unesp-Botucatu sheep acute pain scale; VAS - visual analogue scale. Time points: M1 – 24 h preoperative; M2 – 2 h postoperative, before analgesic rescue; M3 – postoperative, 30 minutes after first analgesic rescue; M4 – 24 h postoperative; MG - data of grouped time points (M1 + M2 + M3 + M4). Statistical analyses were performed using R software (version 4.1.0; 29 June 2021). The functions and packages used are presented in the ‘package:function’ format corresponding to the computer programming language in R. A significant level of 5% was used for all tests. All the figures used a palette of colors distinguishable by people with common forms of color blindness (ggplot2::scale_colour_viridis_d). The database was built with the scores from the two assessment phases of the four observers [23].

## Results

There was neither difference in the number of transoperative fentanyl doses nor post-operative analgesic rescues between the three groups of locoregional anesthesia.

Rescue analgesics were administered to 17 of 23 sheep after M2 video recording because their USAPS total score was ≥ 3. Therefore, only the data of these 17 sheep were used for statistical analysis at M3. A second analgesic rescue was administered to seven sheep 30 minutes later, and a third was performed on one sheep 30 minutes later.

### Distribution of scores

**Fig 2:**
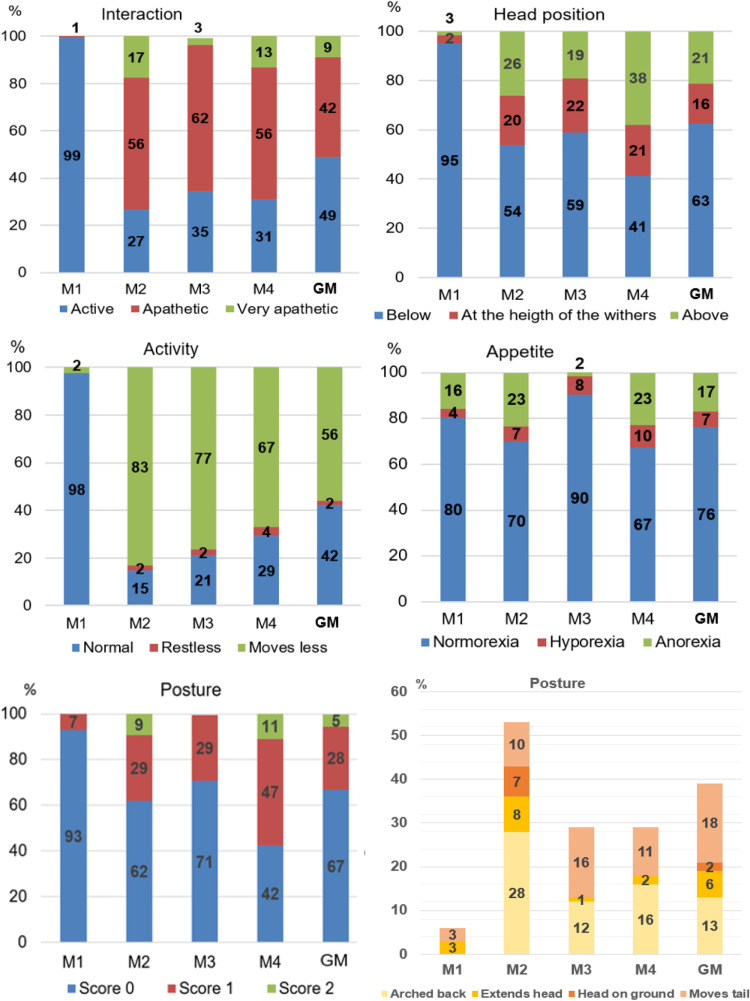
Percentage of the USAPS scores for each item and frequency of each posture subitem. USAPS: Unesp-Botucatu sheep acute pain scale. Time points: M1 – 24 h preoperative; M2 – 2 h postoperative, before analgesic rescue; M3 – postoperative, 30 minutes after first analgesic rescue; M4 – 24 h postoperative; GM – grouped time points data (M1 + M2 + M3 + M4).

The score ‘0’ was mainly present (80–99%) in all USAPS items before surgery (M1). In all items, scores ‘1’ and ‘2’ increased after surgery (M2), decreased after analgesic rescue (M3), and increased again 24h after surgery (M4; except for activity). The most frequent postures after surgery were ‘arched back’ and ‘moves tail’ (Fig 2).

### Multiple association and dimensional structure

**Fig 3:**
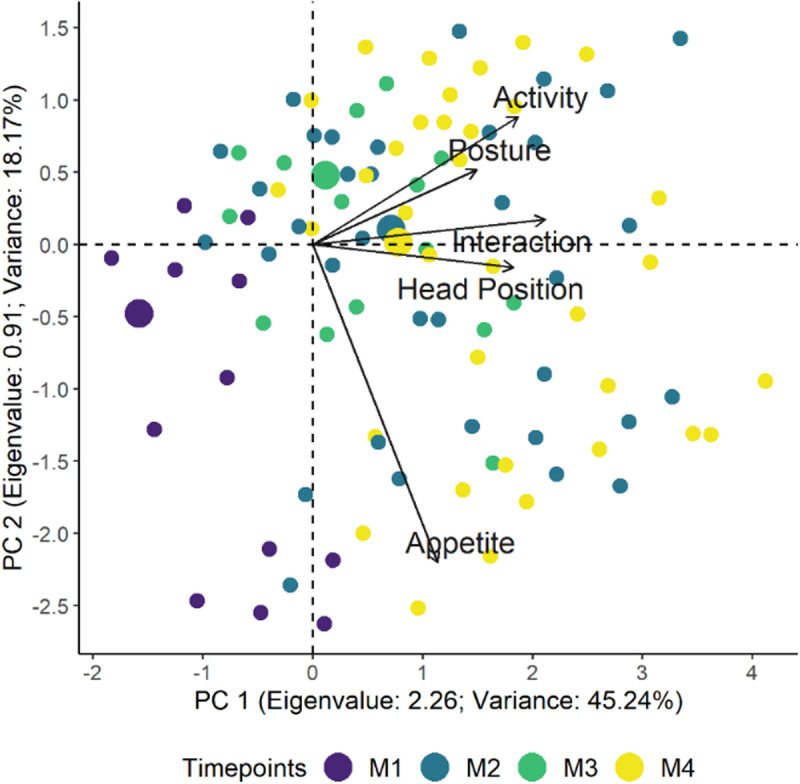
Biplot of the USAPS principal component analysis. Individuals have been placed along the first two principal components (PC) axes and colored according to the time point. Smaller circles indicate each assessment, while larger circles indicate the centroid of each time point and represent the gravitational center of all the vectors when a line is drawn from points of the same color, leading to its geometric center. Therefore, the centroids related to the time points of greatest pain (M2 – after surgery and M4 - 24 h) are positioned to the right, as well as the positioning of the vectors of each item, while the centroid of no pain is positioned to the left (M1- baseline), and the centroid of moderate pain (M3 - rescue) is close to the center of the figure.

Horn’s parallel analysis indicated the retention of the first from the five principal components generated by the principal component analysis. Interaction had a satisfactory loading value in the first principal component, and head position and activity were close (Table 3A; Fig 3). According to the confirmatory factor analysis, the comparative fit values index (0.96) and the Tucker-Lewis index (0.92) values, demonstrate the adequacy of the proposed unidimensional structure; however, the root mean square errors of approximation values were above 0.05 (Table 3B).

**Table 3:** (A) Loading values, eigenvalues, and variance of the USAPS items based on principal components analysis; (B) Confirmatory factor analysis estimates using all USAPS items in a unidimensional structure.

A		
USAPS items	Loading values	
	PC1#	PC2
Interaction	**0.55**	0.07
Head position	0.47	- 0.06
Posture	0.38	0.21
Activity	0.49	0.36
Appetite	0.29	**- 0.90**
**Eigenvalues**	2.26	0.91
**Variance (%)**	45.24	18.17
B		
**Estimates**	**Values**	
Comparative Fit Index	**0.96**	
Tucker Lewis Index	**0.92**	
RMSEA	0.08 (0.05–0.11)	

USAPS: Unesp-Botucatu sheep acute pain scale. (A) PC: principal component 1 and 2 generated by the principal component analysis; # indicate the PC retained for the Horn’s parallel analysis; Items with a loading value ≥ 0.50 or ≤ - 0.50 (highlighted in bold) were considered associated with the PC; (B) Interpretation for Comparative Fit and Tucker–Lewis Indexes: good > 0.90–0.94, excellent ≥ 0.95. Root mean square error of approximation acceptable values < 0.05 [25]. RMSEA: root mean square error of approximation; Only the first PC was retained by Horn’s Parallel analysis [24].

### Intra-observer reliability

The repeatability of the USAPS total score was very good (> 0.81; 0.73–0.95) for all evaluators and ranged from moderate to very good (0.42–0.93) for all items on the scale (Table 4).

**Table 4:** Intra-observer reliability of the USAPS (items, sub-items and total score), visual analogue scale and analgesic rescue indication in the perioperative period of sheep submitted to arthrotomy.

Observer	1			2			3			4		
**Parameter**	Est	Min	Max	Est	Min	Max	Est	Min	Max	Est	Min	Max
Interaction*	**0.75**	0.63	0.85	**0.61**	0.43	0.75	**0.61**	0.41	0.75	**0.69**	0.52	0.8
Head Position*	**0.67**	0.49	0.84	**0.71**	0.55	0.84	0.43	0.2	0.65	0.52	0.34	0.67
Activity*	**0.7**	0.52	0.85	**0.78**	0.64	0.9	0.42	0.24	0.61	**0.8**	0.64	0.93
Appetite*	**0.86**	0.72	0.96	0.54	0.31	0.72	0.44	0	0.8	**0.93**	0.86	0.97
Posture*	0.58	0.38	0.74	**0.61**	0.42	0.79	0.54	0.3	0.73	**0.66**	0.49	0.79
Posture A*	0.53	0.27	0.74	0.59	0.35	0.78				0.33	-0.03	0.66
Posture B*							0.35	-0.04	0.65	0.44	0.12	0.69
Posture C*												
Posture D*				0.59	0.38	0.79	0.58	0.3	0.78	**0.67**	0.49	0.84
**USAPS total score** ^**#**^	**0.93**	0.89	0.95	**0.9**	0.85	0.94	**0.82**	0.73	0.89	**0.9**	0.85	0.94
**VAS score** ^**#**^	**0.85**	0.77	0.9	**0.86**	0.79	0.91	**0.64**	0.45	0.77	**0.89**	0.82	0.93
Analgesic rescue*	0.42	0.11	0.68	**0.72**	0.56	0.85	0.53	0.34	0.71	**0.65**	0.48	0.79

USAPS: Unesp-Botucatu sheep acute pain scale; Est - estimate, Min – minimum, Max – maximum; VAS - visual analogue scale; *Kw - Weighted kappa coefficient; #ICC – intraclass correlation coefficient; Interpretation of reliability: very good 0.81–1.0; good 0.61–0.80; moderate 0.41–0.60; reasonable 0.21–0.4; poor < 0.2 (acceptable values in bold > 0.60) [26,27]

### Inter-observer reliability

The reproducibility of the total USAPS score according to the matrix correlation was very good (ICC: 0.84–0.9) in all inter-observer comparisons, except for two, which were good (ICC of 0.77 and 0.80). Reproducibility ranged from reasonable to very good for all the USAPS items, except for posture, where comparisons between two observers were poor (Table 5).

**Table 5:** Inter-observer reliability of the USAPS (items, sub-items and total USAPS), visual analogue scale and analgesic rescue indication in the perioperative period of sheep submitted to arthrotomy.

Observer	4 vs									1 vs						2 vs		
	1			2			3			2			3			3		
Parameter	Est	Min	Max	Est	Min	Max	Est	Min	Max	Est	Min	Max	Est	Min	Max	Est	Min	Max
Interaction*	0.57	0.46	0.67	0.58	0.45	0.68	**0.66**	0.55	0.74	0.55	0.45	0.63	**0.62**	0.51	0.73	0.48	0.35	0.60
Head Position*	0.54	0.41	0.66	**0.65**	0.53	0.74	0.47	0.33	0.60	**0.62**	0.50	0.73	0.51	0.36	0.64	0.37	0.23	0.50
Activity*	**0.82**	0.72	0.90	**0.64**	0.52	0.75	0.45	0.35	0.57	0.52	0.41	0.61	0.37	0.19	0.52	0.41	0.26	0.55
Appetite*	**0.86**	0.78	0.92	**0.72**	0.60	0.82	0.27	0.12	0.42	0.58	0.46	0.69	0.45	0.35	0.56	0.5.	0.38	0.62
Posture*	0.19	0.04	0.34	0.42	0.27	0.55	0.34	0.20	0.45	**0.73**	0.62	0.84	0.29	0.14	0.44	0.15	0.04	0.28
Posture A*	0.12	-0.04	0.26	0.25	0.07	0.42	0.10	-0.04	0.35	0.42	0.24	0.57	0.11	-0.01	0.23	0.13	-0.01	0.28
Posture B*	0.00	0.00	0.00	-0.03	-0.06	0.00	0.14	-0.04	0.34	NA	NA	NA	0.00	0.00	0.00	-0.03	-0.06	0.00
Posture C*	NA	NA	NA	0.31	-0.03	0.65	-0.03	-0.05	-0.01	NA	NA	NA	NA	NA	NA	NA	NA	NA
Posture D*	0.42	0.27	0.57	0.54	0.38	0.68	0.37	0.20	0.51	0.52	0.33	0.68	0.30	0.10	0.49	0.34	0.16	0.50
**USAPS total score** ^**#**^	**0.90**	0.86	0.92	**0.87**	0.82	0.90	**0.80**	0.73	0.85	**0.89**	0.85	0.92	**0.84**	0.78	0.88	**0.77**	0.69	0.83
**VAS score** ^**#**^	**0.69**	0.58	0.77	**0.8**	0.72	0.85	**0.63**	0.49	0.72	**0.77**	0.69	0.83	**0.65**	0.52	0.74	**0.67**	0.55	0.75
Analgesic rescue*	0.20	0.10	0.30	0.57	0.44	0.68	0.42	0.29	0.54	0.31	0.21	0.42	0.29	0.14	0.44	0.45	0.31	0.57

USAPS - Unesp-Botucatu sheep acute pain scale; VAS - visual analogue scale; *kw - Weighted kappa coefficient; #ICC – intraclass correlation coefficient. Est – Estimate; Min – minimum, Max – maximum. NA – value not available (issues in calculating weighted kappa due to a low variance or identical values). Interpretation of reliability: very good 0.81–1.0; good 0.61–0.80; moderate 0.41–0.60; reasonable 0.21–0.4; poor < 0.20. In bold values that fulfilled the adapted GRADE criteria: acceptable > 0.60) [26,27].

### Criterion validity

Spearman’s coefficient between the USAPS and VAS scores was 0.80 (ICC 0.77–0.83), confirming concurrent criterion validity (rs ≥ 0.80: very strong) [28].

### Construct validity and responsiveness

**Fig 4:**
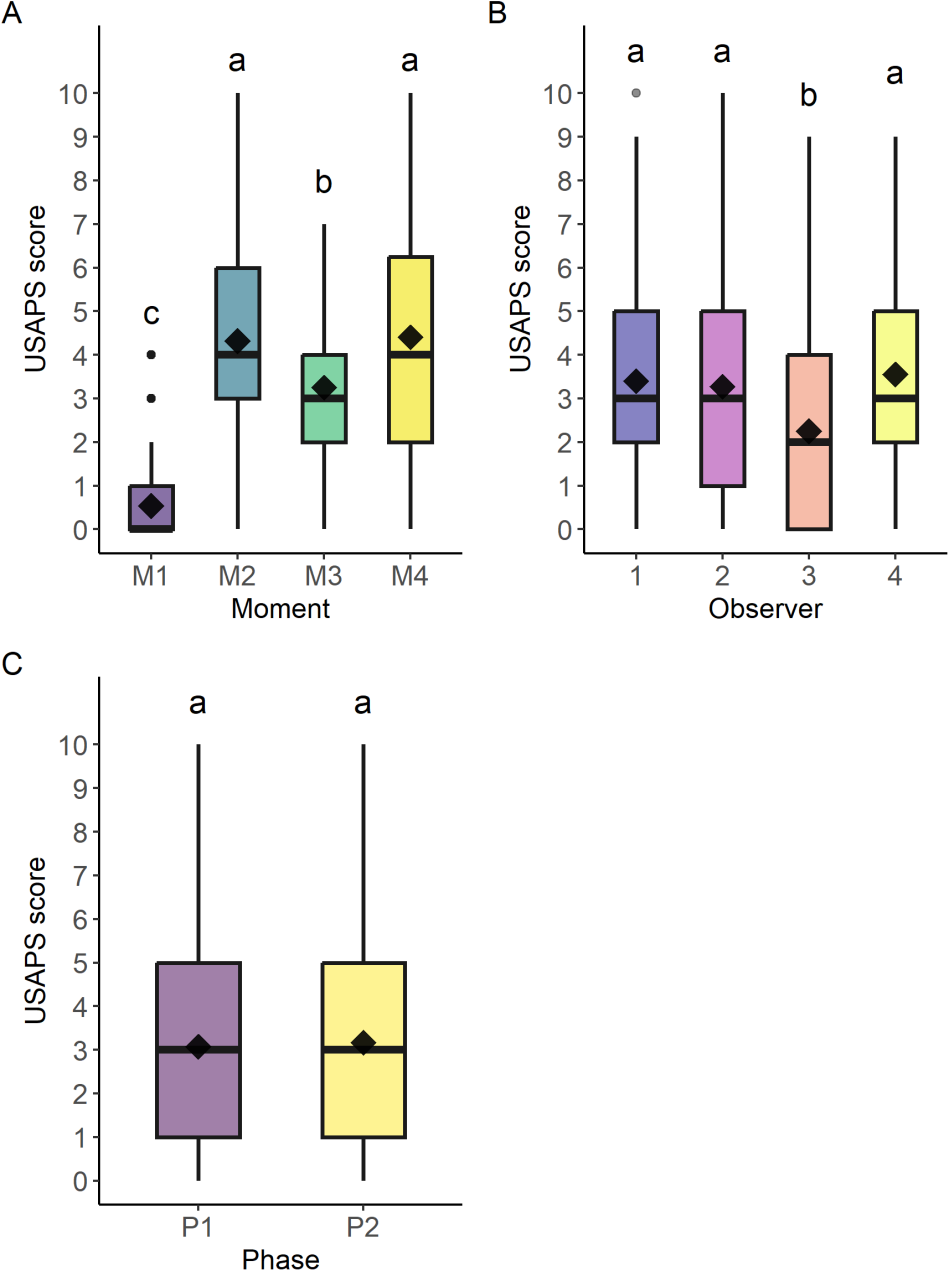
Box plot of the scores (median/amplitude) of the USAPS according to the: (A) time point; (B) observers and (C) phases of evaluation. The top and bottom box lines represent the interquartile range (25 to 75%), the bold line within the box represents the median, the extremes of the whiskers represent the minimum and maximum values, the black diamonds (♦) represent the mean, the black circles (•) represent outliers. Different letters express statistical differences in the Bonferroni test between time points, where a > b > c > d. USAPS - Unesp-Botucatu sheep acute pain scale. Time points: M1 – 24 h preoperative; M2 – 2 h postoperative, before analgesic rescue; M3 – postoperative, 30 minutes after first analgesic rescue; M4 – 24 h postoperative.

Responsiveness was confirmed because the USAPS total score was significantly higher at M2 when the most intense pain was expected after surgery, and M4 at 24h when analgesia was losing its efficacy, followed by M3 when analgesia was provided and then at M1 when sheep were pain-free (Table 6; Fig 4A). Individually, all the USAPS items responded to pain because their scores increased at all times after surgery compared to the baseline value (M1), but only appetite improved after analgesic rescue (M3).

**Table 6:** Responsiveness of the USAPS, VAS and rescue analgesia indication. Time point, observer and phase effects.

Time point	M1		M2		M3		M4		*p-value* from fixed effects		
Parameter	Median	Min-Max	Median	Min-Max	Median	Min-Max	Median	Min-Max	Time p	Observer	Phase
**Interaction**	0^b^	0-1	1^a^	0-2	1^a^	0-2	1^a^	0-2	**< 0.001**	**0.005**	0.258
**Head Position**	0^c^	0-0	0^ab^	0-2	0^b^	0-1	1^a^	0-2	**< 0.001**	0.358	0.726
**Activity**	0^b^	0-2	2^a^	0-2	2^a^	0-2	2^a^	0-2	**< 0.001**	0.654	0.253
**Appetite**	0^c^	0-2	0^a^	0-2	0^b^	0-2	0^a^	0-2	**0.009**	0.130	0.333
**Posture**	0^c^	0-1	0^b^	0-2	0^b^	0-1	1^a^	0-2	**< 0.001**	**0.048**	0.389
Posture A	0	0-0	0	0-1	0	0-1	0	0-1	0.742	**< 0.001**	0.791
Posture B	0	0-1	0	0-1	0	0-1	0	0-1	**0.038**	**< 0.001**	0.078
Posture C	0^b^	0-1	0^a^	0-1	0^ab^	0-1	0^ab^	0-1	**0.007**	**0.006**	0.254
Posture D	0^c^	0-1	0^b^	0-1	0^b^	0-1	0^a^	0-1	**0.006**	**< 0.001**	0.454
**USAPS score**	**0** ^c^	0-4	**4** ^a^	0-10	**3** ^b^	0-7	**4** ^a^	0-10	**< 0.001**	**< 0.001**	0.489
**VAS score**	**2** ^d^	0-50	**22.5** ^b^	0-95	**17** ^c^	0-77	**28.5** ^a^	0-96	**< 0.001**	**< 0.001**	**<0.001**
Analgesia	0^c^	0-1	0^b^	0-1	0^a^	0-1	0^a^	0-1	**< 0.001**	**< 0.001**	0.065

Scales: each item (0–2), sub-items (0–1) and total score (0–10) of the USAPS: Unesp-Botucatu sheep acute pain scale; VAS: visual analogue scale (0–100); Rescue analgesia (0 - no indication; 1 - indication). Data are presented as median (minimum - maximum); Different letters express statistical differences between time points where a > b > c > d. Multiple comparisons were conducted by linear or general mixed models with post-test corrected by the Bonferroni procedure (*p* < 0.05). Bold results represent *p *< 0.05. Time points: M1 – 24 h preoperative; M2 – 2 h postoperative, before rescue analgesia; M3 – postoperative, 30 minutes after rescue analgesia; M4 – 24 h postoperative

**Fig 5:**
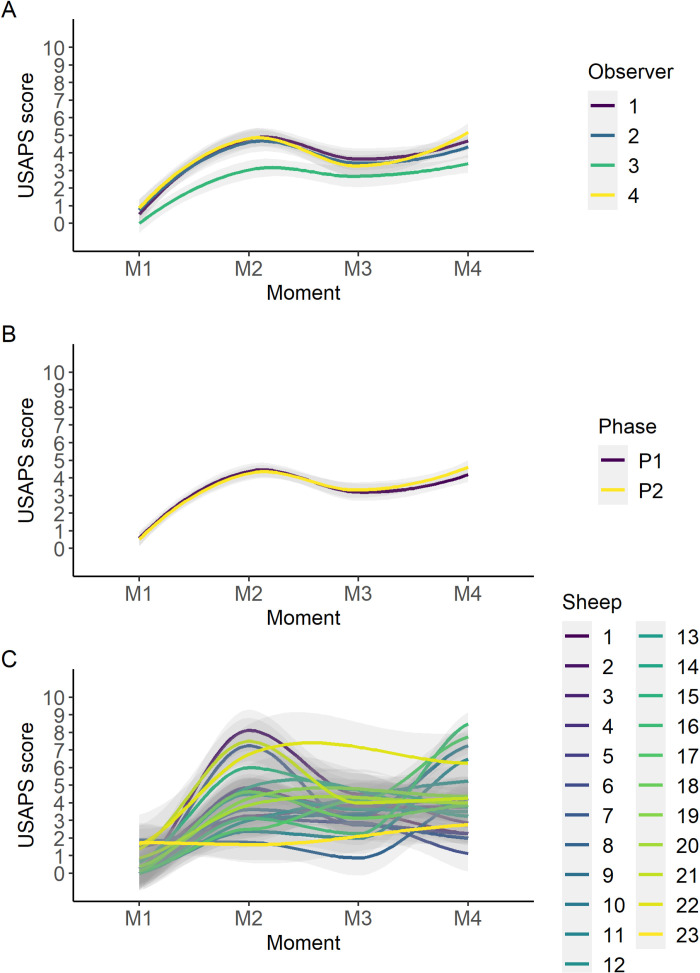
Smooth tendency lines, according to the LOESS method, indicate the scores of USAPS over time points for each evaluator (A), phase (B) and sheep (C). The shaded area corresponds to the standard error of the smooth lines. USAPS: Unesp-Botucatu sheep acute pain scale. Time points: M1 – 24 h preoperative; M2 – 2 h postoperative, before analgesic rescue; M3 – postoperative, 30 minutes after first analgesic rescue and M4 – 24 h postoperative.

The time point as expected influenced all items. There was an observer effect for posture and interaction items. The USAPS total score of observer 3 was lower than the other observers (Fig 4B). Phase did not affect the USAPS total score (Fig 4C) but did affect VAS scores. Considering the total USAPS score over time, there were no variations among observers (Fig 5A) and phases (Fig 5B), but there was variation for each animal (Fig 5C).

### Item-total correlation

The correlation coefficient of each item score with the total score excluding the target item ranged from 0.38 to 0.64, so except for ‘appetite’ (0.25), all items showed acceptable item-total correlation and indicated that they did not inflate the result and were not redundant for USAPS (Table 7).

**Table 7:** Item-total correlation and internal consistency of the USAPS.

Parameters	Item-total (Spearman r_s_)	Internal consistency	
		(Cronbach’s α)	(McDonald’s ω)
**USAPS** (all items)		**0.84**	**0.75**
**Excluding each item below**			
Interaction	**0.64**	0.54*	0.67*
Head position	**0.46**	0.60*	0.72*
Posture	**0.38**	0.64*	**0.75**
Activity	**0.50**	0.61*	0.71*
Appetite	0.25	0.69*	**0.78**

Interpretation of Spearman’s rank correlation coefficient (r_s_): > 0.3 - < 0.7 (acceptable values in bold) [26]. Interpretation of Cronbach’s α coefficient values: 0.60–0.64 minimally acceptable; 0.65–0.69 acceptable; 0.70–0.74 good; 0.75–0.80 very good; > 0.80 excellent (acceptable values in bold ≥ 0.60) [30]. Interpretation of McDonald’s ω coefficient: 0.70–0.84 acceptable and > 0.85 strong evidence [31] * indicates lower α and ω values than those observed for the total set of items when the item was excluded. USAPS: Unesp-Botucatu sheep acute pain scale.

### Internal consistency

McDonald’s ω (0.75) and Cronbach’s α coefficients (0.84) were acceptable, indicating that the scale has excellent internal consistency (Table 7). Except for ‘interaction,’ the internal consistency was minimally acceptable (Cronbach’s α) or acceptable (McDonald’s ω) when each item was excluded. Considering that the internal consistency value decreased by excluding all items for α and in eight of 10 items for ω, they contributed significantly to the scale’s total score.

### Specificity and sensitivity

Most of the USAPS items showed good to excellent specificity (83–99%). The interaction and activity items showed moderate or good sensitivity (82–87%), but the other three items were not sensitive (50–67%). The total USAPS score showed maximum specificity and moderate sensitivity (Table 8).

**Table 8:** Specificity and sensitivity and its 95% confidence interval of the USAPS.

Parameters	Specificity (%)			Sensitivity (%)		
	Estimated	Lower	Upper	Estimated	Lower	Upper
Interaction	**99**	95	100	**82**	76	87
Head Position	**91**	82	96	67	61	73
Posture	**83**	72	91	63	57	69
Activity	**97**	93	99	**87**	82	92
Appetite	60	49	71	59	52	65
**USAPS** total score	**100**	96	100	**71**	65	77

Interpretation of specificity and sensitivity: excellent 95–100%; good 85–94.9%; moderate 70–84.9%; not specific or sensitive < 70 [26]. USAPS: Unesp-Botucatu sheep acute pain scale.

### Optimal cut-off point

**Fig 6:**
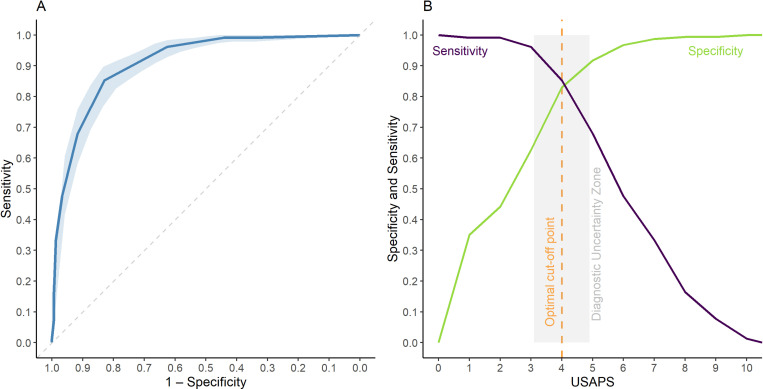
ROC curve and area under the curve (AUC). (A) and two-graph ROC curve with the diagnostic uncertainty zone of the USAPS (B). USAPS - Unesp-Botucatu sheep acute pain scale; ROC (receiver operating characteristic) curve with a 95% confidence interval (CI) calculated from 1,001 replications applied to estimate the diagnostic uncertainty zone of the cut-off point of all evaluators, according to the Youden index for the USAPS. Interpretation of AUC ≥ 0.90 - high discriminatory capacity [32,33]. The diagnostic uncertainty zone was 3.1 - 4.9; ≤ 3 indicates pain-free animals (true negative) and ≥ 5 indicates animals suffering pain (true positive). The Youden index was ≥ 4, representative of the cut-off point for the indication of analgesic rescue, even excluding appetite.

The cut-off point for diagnosing pain and recommending analgesia determined by the ROC curve was 3.5 (3.5–3.5) for USAPS with or without appetite and 26 (21.5–27.5) for VAS. Therefore, the optimal cut-off point is ≥ 4 (out of 10). Based on the diagnostic uncertainty zone between 3.1 and 4.9, a USAPS total score of ≤ 3 indicates sheep without pain (true negatives) and ≥ 5 indicates sheep expressing pain (true positives). The cut-off point had 83% (80–86) of specificity or 86% (82–89), and 85% (81–90) of sensitivity or 81% (77–87) excluding appetite respectively. The area under the curve of 91% (89–93) or 90% (88–93) excluding appetite, indicates that the USAPS has a high discriminatory capacity (Fig 6) [26].

The percentage of animals within the diagnostic uncertainty zone ranged from 1% (in M1) to a maximum of 18% (in M3) (Table 9).

**Table 9:** Percentage of sheep present in the diagnostic uncertainty zone of each observer at each time point for USAPS.

Observer	Time point				GM
M1	M2	M3	M4
1	0	2	24	17	15
2	2	20	18	15	13
3	0	13	15	13	10
4	2	9	15	11	9
**All**	1	15	18	14	12

Calculation based on 23 sheep evaluated twice by 4 observers. Data are presented as the percentage of USAPS scores = 4. All: 4 observers; GM - grouped time points data. Time points: M1 – 24 h preoperative; M2 – 2 h postoperative, before rescue analgesia; M3 – postoperative, 30 minutes after first rescue analgesia; M4 – 24 h postoperative. USAPS: Unesp-Botucatu sheep acute pain scale.

## Discussion

The Unesp-Botucatu sheep acute pain scale (USAPS) applied to an orthopedic pain model is a reliable and valid pain assessment instrument according to COSMIN guidelines, as demonstrated for abdominal surgery, confirming our hypothesis. The cut-off point for analgesic intervention is the same as the original study performed on abdominal pain [9], even excluding the locomotion and appetite items.

The USAPS items’ pain-altered scores (1 and 2) were proportionally distributed following the expected pain level for each perioperative time point (M2 > M4 > M3 > M1), indicating the importance of three levels of scores in each item. The exception was the restless behavior for activity item, which occurrence was low like in the original scale [9]. Restless behavior is also low in scales developed for other species, such as cats [34], cattle [35,36], and horses [37,38]. Although it is rare for a sheep to show more movement than usual or to lie down and get up frequently when experiencing pain, this behavior was classified among the five most important ones according to a previous study [39].

Opposite to expected, sheep submitted to soft tissue surgery had higher pain scores (8; 0–12) than after orthopedic surgeries (4; 0–10). This might be explained by the fact that the abdominal study [9] there simulated a field procedure using minimally invasive video laparoscopy in sheep undergoing dissociative anesthesia, opioid and a short-duration local block. In contrast, in the present study, surgery was more invasive and prolonged; arthrotomy was performed with a multimodal anesthetic and analgesic protocol, including either epidural or perineural block with bupivacaine. The anesthetic effect of the perineural administration of 0.5% bupivacaine lasts 110.0 ± 47.3 minutes [40] and the analgesic duration of epidural administration of 0.25% bupivacaine lasts 240 minutes [41]. In addition, the pain was still present 24h after surgery in the present study, when the effect of bupivacaine had been diminished, otherwise, the median USAPS total score was 0 at 24 h after abdominal surgery [9]. Interestingly, the posture item, moving tail and arched back were the most predominant behaviors after orthopedic surgery against extended head and neck and lying down with the head resting on the ground or close to the ground after abdominal surgery, highlighting the importance of testing the pain assessment instrument with different pain models. Another reason for the lower postoperative scores in the present study than in the previous one [9] was that locomotion was not measured in sheep submitted to orthopedic surgery, therefore the maximal score was 10 instead of 12 [9].

Multiple association tests evaluated the main components of the scale that encompass a group of items within the same principal component (dimension) [24,25]. Although the appetite item was loaded into the second principal component, it was not retained. Therefore, the USAPS for orthopedic surgery showed a unidimensional structure, like previously seen for the first validation of USAPS and the scales developed in cattle [35], goats [42] pigs [43], piglets [44], rabbits [45] and donkeys [46]. From a mathematical point of view, a unidimensional structure allows the sum of all USAPS items into a singular USAPS total score with a singular cut-off point, which makes the pain scale easier to apply compared to multidimensional pain scales. Nevertheless, from a biological point of view, USAPS for orthopedic surgery is multifaceted because it includes sensory, motor, emotional, temporal, and physiological attributes.

Repeatability (intra-observer reliability) and reproducibility (inter-observer reliability) of USAPS for orthopedic surgery were very good and greater than the original scale [9], and a scale developed for lambs undergoing acute pain [10], and similar to a sheep locomotion scoring scale [47]. Concurrent criterion validity assesses the measurement efficiency of a scale by comparing it with a validated instrument. Because there is no other gold standard validated sheep pain scale other than USAPS [13], we used the same approach performed in the previous study by comparing USAPS with VAS. The USAPS correlated strongly with VAS [28] like for soft tissue surgery [9]. Another way of confirming concurrent criterion validity was the very good agreement between most evaluators. Predictive criterion validity was confirmed by sensitivity, as 71% of sheep would receive analgesia based on the cut-off point at the time point of most intense pain (M2).

Responsiveness confirmed the ability of USAPS to distinguish differences in pain scores over time points by detecting the effect of pain and analgesic intervention [26]. All USAPS items increased in response to pain, but their scores were little affected by analgesia because the pre and transoperative analgesic protocol, including bupivacaine, produced preventive analgesia after orthopedic surgery compared to abdominal one [9], where local anesthesia was performed with 1% lidocaine, a short-lasting local anesthetic. In fact, six sheep did not require analgesia after arthrotomy. Another explanation for the lower pain score after arthrotomy is the different anesthetic protocol (diazepam/ketamine and epidural anesthesia with 1% lidocaine for laparoscopy against a more complex multi-balanced anesthesia and analgesia protocol for arthrotomy, including one transoperative injection of fentanyl in seven sheep. Indeed, in the current study, pain scores were still high at 24h, like those after surgery, even considering that six sheep received two and one received three additional analgesic rescues. Although laparoscopy does cause acute inflammation like arthrotomy, when the effect of analgesia wore off, we hypothesized that the dysfunctional component triggered by meniscectomy added a more intense nociceptive stimulus at 24h.

Construct validity was confirmed by testing three hypotheses: 1. the postoperative pain scores were higher than the preoperative score (M1 > M2), 2. the scores decreased after analgesia (M2 > M3) and 3. the scores increased again by 24h when the effect of analgesia faded (M2 ≥ M4 > M3 > M1)], known-groups validity, internal relationships among items (internal consistency, item-total correlation and principal component analysis) and relationships to scores of other instruments (strong Spearman correlation between USAPS and VAS).

Most USAPS behaviors may increase when sheep experience pain and after sedation. However, both USAPS and VAS pain scores reduced 30 minutes after analgesia (M3) compared to M2 (after surgery), therefore sheep were apparently experiencing pain in M2. Otherwise, if USAPS measured sedation, it would be more likely that pain scores would remain high in M3 due to sedation (higher scores of interaction, locomotion, head position and activity). Seven of 17 sheep received metamizole, 30 minutes after the first analgesic rescue with meloxicam and morphine. According to the Wilcoxon test the median score of these seven sheep reduced significantly from 3 (0–7) after meloxicam and morphine to 2.5 (0–7) after metamizole (p = 0.002), showing that scores were responsive to a drug which does not affect sedation.

The USAPS posture and interaction items and total score were susceptible to an observer effect, as reported in caprine [42] and leporine [45], however, in the present study, the differences among observers were caused by only one observer. Phases did not affect USAPS, but affected VAS. The individual variability in pain scores was expected because the expression of pain is individual, as reported in bovine [36].

The item-total correlation analyses the homogeneity of the scale items. Since the item-total correlation ranged between 0.30 and 0.70, the items were not redundant when analyzed singularly, contributing to and correlating well with the USAPS total score [26]. The only exception was the appetite.

The scale had excellent internal consistency like for abdominal pain [9]. When each of the five items was excluded, the internal consistency decreased, indicating that all items were relevant [30]. The only exception was the McDonald’s ω coefficient for appetite. Cronbach’s α coefficient is the most used method for assessing internal consistency, however, it assumes that items have equal covariances. To overcome this limitation, McDonald’s ω coefficient [31], was applied, and in this case, internal consistency was acceptable (ω = 0.75).

All USAPS items, except appetite, were specific, and the USAPS total score showed maximum specificity (100%). Three of five USAPS items did not show sensitivity (< 70%): appetite, posture, and head position. Appetite and posture did not show sensitivity in the laparoscopy study either [9]. The scale identifies 100% of animals with no pain (before surgery; scores < 4), preventing unnecessary treatment [26]. However, the scale´s ability to identify animals experiencing pain was moderate (71%), which increases the possibility of an observer not providing analgesic intervention when necessary. One possible explanation for this is that the sensitivity calculation was performed two hours after surgery when the effect of multimodal analgesia was still present, thus preventing the animals from fully expressing pain by this time.

The ROC curve estimated the optimal cut-off point for diagnosing pain and recommended analgesia with good sensitivity and specificity. The AUC of 91% indicated the high diagnostic accuracy of USAPS in distinguishing sheep with or without pain [33].

The cut-off points for indication of analgesia for VAS (≥ 26 of 100) and USAPS (≥ 4 of 10), even without locomotion, were the same as reported for abdominal surgery (≥ 4 of 12) [9]. Appetite item exclusion did not affect the cut-off point. Therefore, this is extra evidence that appetite could be removed without further impairment in the pain diagnosis.

The appetite item was not within the acceptable values for item-total correlation, internal consistency (McDonald’s ω), sensitivity, and specificity, as in the previous study [9] and considering that the cut-off point was not affected by the exclusion of appetite in the previous study [9] and in the current one, it may be excluded from USAPS.

The selection and training of observers are important to ensure reliability in assessing pain [27,48]. Reliability improved when experienced observers assessed pain in horses and piglets compared to veterinary students [49] or less experienced observers respectively [50]. Students tend to underestimate pain in bulls, cats, pigs and sheep compared to an expert [51] and training improved pain recognition in laboratory animals [48,52]. The observers of the current study had previous experience in assessing pain scales in other species. Two of them (FAO and MOF) participated in the original study [9] and all became familiar with the USAPS behaviors by training on the https://animalpain.org/en website (Table 1). They all had intra and inter-rater reliability ≥ 80% after a pretraining phase before scoring USAPS in the main study, which may have favoured the reliability results. Therefore, future research should compare trained versus untrained observers in scoring USAPS.

In the current study, the videos were sent to the observers weekly for a gradual evaluation, to minimize fatigue [45]. USAPS and VAS were assessed in a random order. There are pros and cons of this approach. In our previous studies, the VAS was assessed first to prevent the expectation bias of the behavioral scales from influencing the VAS [9]. Even with this approach, for all observers VAS intra, and inter-rater reliabilities were worse than USAPS, and there was a phase-effect in responsiveness, reinforcing that the behavioral scale is more reliable for assessing pain than VAS, like in cats [53], horses [38], donkeys [46] and rabbits [45].

We demonstrated that implementing statistical weighting based on machine learning algorithms can rank the importance of each USAPS pain-related behavior to identify sheep requiring or not analgesia and improve the diagnostic capacity based on the weighted USAPS [39] compared to the original one [9]. Pain-specific behaviors occurred almost exclusively at the time of greatest pain (M2) and less throughout the other post-operative period. Otherwise, as expected, maintenance behaviors, like activity, locomotion, and interaction, reduced after surgery (M2) [9]. When the USAPS pain-related behaviors were weighted [39], maintenance behaviors were more important for pain diagnosis than the increase in the frequency of specific pain-related behaviors.

The current study has some limitations. There was not another gold standard pain scale to compare against USAPS and better assess concurrent criterion validity. As in the previous study [9], only adult female sheep were assessed. Specifically in lambs, female lambs displayed more pain avoidance behaviors (sum of all) after tail docking than male lambs. In alpha rams, USAPS showed satisfactory sensitivity and specificity for diagnosing pain after orchiectomy [54].

Observers’ presence can inhibit pain expression, increasing the possibility of false negative results [55,56]. The real-time assessment had comparable results to video observation of the Unesp-Botucatu piglet [57] and cattle [58] acute pain scales. However, because remote camera visualization is not commonly available in routine clinical and field situations, we opted to maintain the researcher present to ensure that results are reproducible in a real-world scenario.

Although we did our best to blind the observers, the animals were lame in the left limb after the orthopedic surgery. Therefore, even considering the randomized order of all videos and animals, the observers might recognize the preoperative period videos, but not the other three postoperative time points.

In the future, it is important to evaluate the scale’s psychometric properties in other languages and cultures, like performed in cats [59] and cattle [35,36,60]. Another limitation of the study is the possible influence of the circadian cycle on sheep pain behavioral expression [61], as reported in horses [62], as they reduce activity at night. The current study occurred in the summer and daylight was present at all periods sheep were assessed. Therefore, other studies could test USAPS during nighttime.

Behavior changes caused by sedation and pain may be similar, therefore sedation may be a confounding factor when assessing pain in all species and may increase the chance of a false positive pain diagnosis. Although the residual effect of drugs used in the current study was minimal, considering their half-life and the late first postoperative measurement time, a future study should be performed, with a negative analgesic control group treated only with sedation, to test only the effect of sedation in USAPS.

## Conclusion

The USAPS scale has a high strength of evidence following the COSMIN guidelines to diagnose pain in sheep undergoing arthrotomy. It has satisfactory psychometric properties and therefore can be applied for assessing pain in sheep models of orthopedic surgery. The cut-off point is just a guide to improve decision-making on whether to provide analgesia, however, the final judgment should be based on clinical experience and context, to ensure that sheep suffering pain would be treated accordingly.

## Supporting information

Table S1 Grading of Recommendations, Assessment, Development, and Evaluations (GRADE). (DOCX)

Table S2 Animal Research Reporting In Vivo Experiments (ARRIVE 2.0). (PDF)

S1 Appendix Dataset used for this study. (XLSX)
