# Supplementary material for: Clinical validation of the Unesp-Botucatu acute pain scale in sheep undergoing orthopedic surgery
Source: PLoS One. 2025 May 12;20(5):e0323132. doi: 10.1371/journal.pone.0323132 (PMC12068710; doi:10.1371/journal.pone.0323132)
Supplement: Table S1 — (DOCX) [file pone.0323132.s001.docx]

**Table S1.** Psychometric score of GRADE adapted to test the properties of the USAPS

| **Questions** | **Subtitle score** | **USAPS ortho (current study)** | **USAPS abdom (original study)** |
| --- | --- | --- | --- |

**Scale development: item selection and content validation**

| **1.1. Was the process of item selection described?** | 2: Scale was developed for a specific population, using a theoretical or conceptual framework, or a qualitative approach was used (e.g., consultation with clinicians or patients)  1: Scale was developed based on the literature review only  0: No information is provided about item selection | 2 | 2 |
| --- | --- | --- | --- |
| **1.2. Was content evaluated by experts? (content validation)** | 2: Content was evaluated by experts in the field, a Delphi technique may have been used, and Content Validity Index (CVI) were calculated for each item included in the scale  1: Content was evaluated by experts, but no CVI is reported  0: No information is provided about content validation | 1 | 1 |
| **1.3. Are limitations of some items presented or discussed?** | 1: No limitations or if any limitations, they are presented and item modifications have  been made or precautions have been stated 0: No information is provided | 1 | 1 |
| **Subtotal-Scale Development (0 – 5)** | | 4/5 | 4/5 |
| **Subtotal weighted score—Scale development (0–2)** | | **1.6** | **1.6** |

**Scale testing-reliability**

| **2.1. Was internal consistency of the scale calculated?** | 2: 0.70 < α< 0.90  1: 0.60 < α < 0.70 or α> 0.90 0: α < 0.60 or no  information provided | 2 (α = 0.84; ω = 0.75) | 2 (α = 0.81) |
| --- | --- | --- | --- |
| **2.2. Was interrater reliability calculated?** | 2: kappa > 0.60 or ICC > 0.80  1: 0.60 < kappa > 0.40 or 0.60 < ICC < 0.80  0: kappa < 0.40, ICC < 0.60 or no information provided | 1 (ICC ≥ 0.77) | 0 (ICC ≥ 0.52) |
| **2.3. Was interrater reliability tested**  **with other raters besides research team?** | 1: Other raters then research staff members were involved | 1 | 1 |

|  | 0: Only research staff members were involved |  |  |
| --- | --- | --- | --- |
| **2.4. Was intrarrater reliability tested? Optional—to be examined if ICC <**  **0.80 for interrater reliability** | 2: kappa > 0.60 or ICC > 0.80  1: 0.60 < kappa > 0.40 or 0.60 < ICC < 0.80  0: kappa < 0.40, ICC < 0.60 or no information provided | 2 (ICC ≥ 0.82) | 1 (ICC ≥ 0.65) |
| **Subtotal—Scale development (0–5 or 0–7 if intrarrater reliability testing required)** | | 6/7 | 4/7 |
| **Subtotal weighted score—Scale development (0–6)** | | **5.14** | **3.42** |

**Scale testing: Construct validity**

| **3.1. What is the total of participants for the purpose of testing the scale?** | 2 – N > 50  1 – 20 > N < 50  0 – N < 20 | 1 | 1 |
| --- | --- | --- | --- |
| **3.2. Criterion validation: Was the scale correlated with the “gold standard: measure renowned in the field of interest (e.g., the patient’s self-report of pain)?** | 2: r > 0.60 with the “gold standard” measure  1: 0.40 < r < 0.60  0: r < 0.40 or no information provided | 2 (r = 0.80)  (VAS was considered "gold standard") | 2 (r = 0.81)  (VAS was considered "gold standard") |
| **3.3. Criterion validation: Was the sensitivity of the scale calculated?** | 2: Sensitivity ≥80% 1: 60% Sensitivity < 80%  0: Sensitivity < 60% or no information provided | 2 (S = 1) | 1 (S > 0.61) |
| **3.4. Criterion validation: Was the specificity of the scale calculated?** | 2: Specificity ≥ 80%  1: 60% ≤ Specificity < 80%  0: Specificity < 60% or no information provided | 1 (Sp > 0.70) | 1 (Sp > 0.72) |
| **3.5. Discriminant validation: Was the scale able to discriminate between different situations (e.g., between pain and no pain, e.g., at rest and during a nociceptive procedure,**  **before and after the administration of an analgesic)?** | 2: A clinically important difference was found  1: A difference was found but was not considered clinically important  0: No difference was found or no information is provided | 2 | 2 |
| **Subtotal—Scale development (0–10)** | | 8/10 | 6/10 |
| **Subtotal weighted score—Scale development (0–8)** | | **6.4** | **4.8** |
| **4.1. Was the feasibility (i.e., ease of usage with which clinicians can apply the instrument in the clinical setting) of the scale examined?** | 1: Scale is considered to be feasible to use by more than 80% of the clinicians  0: Scale is considered to be complex to use  by more than 20% of the clinicians or no information is provided | 0 | 0 |

| **4.2. Are directives of use of the scale clearly described?** | 1: Yes, directives of use including the scoring method are described  0: No information about directives of use is provided | 1 | 1 |
| --- | --- | --- | --- |
| **Subtotal—Scale development (0–2)** | | 1/2 | ½ |
| **Subtotal weighted score—Scale development (0–2)** | | **1** | **1** |
| **5.1. Was the relevance of the scale or impact of its implementation in patient outcomes examined?** | 1: Scale is considered to be useful and relevant to practice by more than 80% of the clinicians; use of the scale yielded a significant change into practice (e.g., better use of medication, increase in patients’ assessments)  0: Scale is not considered to be useful and relevant to practice by more than 20% of the clinicians; use of the scale did not yield a significant change into practice or no information provided | 0 | 0 |
| **Subtotal—Scale development (0–1)** | | 0/1 | 0/1 |
| **Subtotal weighted score—Scale development (0–2)** | | **0** | **0** |
| **Total score (0–25)** | | 19 | 15 |
| **Weighted score (0–20)** | | **14,14** | **10,82** |
| **Quality of evidence** | | Good | Moderated |

Interpretation: Weighting scores 15 a 20 – Very good; 12 – 14.9 – good; 10-11.9 – Moderated; 0 – 9.9 – Few/unacceptable properties. Scales with weighting ≥ 12 showed greater validity and reliability
